# The impact of hormone receptor on the clinical outcomes of HER2-positive breast cancer: a population-based study

**DOI:** 10.1007/s10147-022-02115-x

**Published:** 2022-01-18

**Authors:** Yiqun Han, Yun Wu, Hangcheng Xu, Jiayu Wang, Binghe Xu

**Affiliations:** Department of Medical Oncology, National Cancer Center/National Clinical Research Center for Cancer/Cancer Hospital, Chinese Academy of Medical Sciences and Peking Union Medical College, No. 17, Panjiayuan Nanli, Chaoyang District, Beijing, 100021 China

**Keywords:** Breast cancer, HER2-positive, Hormone receptor, Clinical features, Prognosis

## Abstract

**Background:**

To investigate the impact of hormone receptor (HR) on the clinicopathological characteristics and prognosis of human epidermal growth factor receptor 2 (HER2)-positive breast cancer.

**Methods:**

Using the Surveillance, Epidemiology, and End Results database, we enrolled patients diagnosed with HER2-positive breast cancer between 2010 and 2016, which were successively assessed for eligibility and categorized into HR + /HER2 + and HR-/HER2 + subgroups. Clinicopathological characteristics were undergone comparative analyses with the baseline distinctions calibrated by propensity score matching, while the survival outcomes were compared using Kaplan–Meier method with log-rank tests.

**Results:**

A total of 46,803 HER2-positive breast cancer patients were identified, of which 32,919 individuals were HR + /HER2 + subtype and 13,884 individuals were HR-/HER2 + subtype, respectively. Comparatively, HR + /HER2 + breast cancer presented a lower histological grade, a smaller tumor size, a lower nodal involvement, and a lower rate of de novo stage IV disease. Substantial heterogeneity was detected in the metastatic patterns of organ-specific involvement between the two subgroups with initial metastasis. Overall, patients with HR + /HER2 + tumors had increasingly favorable prognosis in terms of overall survival and breast cancer-specific survival than patients with the HR−/HER2 + subtype. However, this kind of tendency exhibited disparities associated with HR-specific subtypes based on estrogen receptor (ER) and progesterone receptor (PgR) status, in which ER−/PgR + tended to present the worst prognosis.

**Conclusion:**

This study revealed profound heterogeneity associated with HR status in the clinical outcomes of HER2-positive breast cancer regarding clinicopathological features, metastatic patterns, and prognosis. Prospective studies to optimize therapeutic strategies for HER2-positive subgroups are warranted.

**Supplementary Information:**

The online version contains supplementary material available at 10.1007/s10147-022-02115-x.

## Introduction

Breast cancer is the most common malignancy among women, with over 276,000 newly diagnosed cases and nearly 42,000 deaths annually in the United States [1]. Inherently, breast cancer is a heterogeneous disease that is diverse in terms of molecular and clinicopathological characteristics, indicative of potentially different prognosis. According to the expression of human epidermal growth factor receptor 2 (HER2), estrogen receptor (ER), and progesterone receptor (PgR), breast cancer can be classified into four distinctive molecular subtypes recommended as solid reference to the precise treatment and prognosis estimation [2], of which HER2 over-expression is observed in approximately 20–25% of invasive breast cancer and is associated with an inclination of early recurrence and distant metastasis suggestive of an inferior prognosis [3].

Hormone receptors (HR), consisting of ER and PgR, are critical markers for treatment introduction of breast cancer [4]. It was acknowledged that HR-positive breast cancer, compared with HR-negative subgroup, tended to be decreasingly aggressive and of ameliorated prognosis due to endocrine therapy. Previous researches have managed to underline the differences in clinical characteristics, therapeutic responses, and prognosis of HER2-positive breast cancer varied by HR status [5–8]. A retrospective analysis analyzing 450 HER2-positive breast cancer patients showed that HR-negative subtype was associated with an inferior prognosis [9], while some studies indicated no significant difference existing in the long-term survival of patients between the two subgroups [10–12]. To date, given the potentially insufficient sample volume and distinct study design, the clinical impact of the HR status on the prognosis of HER2-positive breast cancer remains controversial.

In this study, based on the large-scale population, we systematically assessed the heterogeneities in the clinical outcomes, regarding clinicopathological features, metastatic patterns, and overall prognosis, of HER2-positive breast cancer associated with HR status. This knowledge was anticipated to provide an opportunity to better understand the tumor behavior and further define personalized treatment strategies for HER2-positive breast cancer patients in clinical practice.

## Materials and methods

### Patient selection

Data on the breast cancer patients were obtained from the Surveillance, Epidemiology, and End Results (SEER) database (2010–2016, November 2018 submission). Since HER2 status and organ-specific involvement were not registered until 2010, this study adopted the cohort dataset for which the initial diagnosis occurred after 2010. Patients were included of which the information of receptor status was complete and ruled out if the demographics and clinicopathological features were missing.

We comprehensively extracted the population demographics and clinicopathological features for each case, consisting of age at diagnosis, race, histologic type, grade, tumor size, nodal involvement, distant metastasis, HR status, HER2 status, surgical performance, radiation treatment, and chemotherapeutic delivery. Due to the publicly accessible nature of the database, this population-based analysis was exempted from approval by the ethics committee of the Chinese Academy of Medical Sciences. The current study was implemented in accordance with the Strengthening the Reporting of Observational Studies in Epidemiology (STROBE) guidelines [13] and the Transparent Reporting of a Multivariate Prediction Model for Individual Prognosis or Diagnosis (TRIPOD) statement [14].

### Variable definition

In this study, HER2-positive breast cancer was classified into the HR + /HER2 + and HR−/HER2 + subgroups with stratification by HR status. HR-specific subtypes comprised ER + /PgR + , ER + /PgR−, ER−/PgR + , and ER−/PgR− subgroups. ER and PgR positivity were defined as ≥ 1% staining. Metastatic patterns were referred to the metastatic status based on the organic involvement which included bone, lung, liver, brain in accordance with the SEER terminology. Overall survival (OS) was defined as the interval from the diagnosis of breast cancer to the death caused by any reason or the last follow-up, while breast cancer-specific survival (BCSS) was the period between the initial diagnosis and cancer-related death. The American Joint Committee on Cancer 7^th^ edition guidelines were adopted to define the TNM staging of breast cancer.

### Statistical analysis

Categorical variables, consisting of population demographics, clinicopathological characteristics, and metastatic patterns, were compared using *χ*^2^ tests and Fishers’ exact probability tests, while continues variables were undergone comparative assessment using *t*-test for normal distribution and Mann–Whitney *U* test for abnormally distributed variables. Survival outcomes were compared using Kaplan–Meier method with log-rank tests. Propensity score matching (PSM) was performed to further evaluate the effect of HR status on survival by eliminating objective distinctions among baseline characteristics between two subgroups of HER2-positive breast cancer. Statistical significance was set as a two‐sided *P* value less than 0.05. For the comparison of four categories, statistical significance was determined by *P* < 0.05/6 using Bonferroni correction to avoid multiplicity. All the statistical analyses were performed using SPSS version 26.0 (Armonk, NY, IBM Corp) and R software 3.6.4.

## Results

### Clinicopathological characteristics

A total of 46,803 HER2-positive breast cancer patients diagnosed from 2010 to 2016 were identified, of which 32,919 (70.3%) were HR + /HER2 + and 13,884 (29.7%) were HR-/HER2 + subtype. Median follow-up time for all enrolled patients was 38 months (range 0–83 months). Duration of follow-up was longer in the HR + group (median 38 months) compared to the HR-group (median 37 months) (*P* = 0.011). The demographics and baseline characteristics of enrolled cohort were summarized in Appendix Table 1. The median age was 58.28 years of the HR + /HER2 + and was 58.11 years of the HR−/HER2 + subgroup patients (*P* = 0.227), respectively. The proportion of the white race was relatively higher in patients with HR + /HER2 + breast cancer (77.1% vs. 72.3%, *P* < 0.0001). Regarding histologic type, HR−/HER2 + tumors predisposed to be diagnosed with invasive ductal carcinoma, while the percentage of invasive lobular carcinoma was significantly higher in HR + /HER2 + subtype breast cancer (*P* < 0.0001). Compared to HR−/HER2 + subtype, HR + /HER2 + breast cancer had a lower tumor grade (III-IV, 51.5% vs. 74.4%, *P* < 0.0001), a smaller tumor size (T0-1, 49.8% vs. 43.4%, *P* < 0.0001), a lower nodal involvement (N0, 60.0% vs. 55.0%, *P* < 0.0001), and a lower rate of de novo stage IV disease (M1, 6.3% vs. 8.0%, *P* < 0.0001). With regard to therapeutic options, patients with HR + /HER2 + breast cancer were more likely to receive surgery (91.2% vs 89.4%, *P* < 0.0001) and radiotherapy (45.0% vs 42.0%, *P* < 0.0001) compared with HR-/HER2 + patients, while the delivery rate of chemotherapy was relatively lower in the HR + /HER2 + subgroup patients (71.5% vs. 76.6%, *P* < 0.0001).

**Table 1:** Comparative analysis of population demographics and baseline characteristics between HR + /HER2 + and HR-/HER2 + subgroups

Characteristics	HR + /HER2 + (*N* = 32919)		HR-/HER2 + (*N* = 13884)		*P* value
	No	Percent (%)	No	Percent (%)	
Age at diagnosis	58.28		58.11		0.227
Race					< 0.0001
White	25397	77.1	10043	72.3	
Black	3844	11.7	1976	14.2	
Others	3678	11.2	1865	13.5	
Histologic type					< 0.0001
Ductal	28270	85.9	12463	89.8	
Lobular	3588	10.9	702	5.1	
Others	1061	3.2	719	5.2	
Grade					< 0.0001
Grade1	2243	6.8	217	1.6	
Grade2	13740	41.7	3346	24.1	
Grade3	16815	51.1	10202	73.5	
Grade4	121	0.4	119	0.9	
T					< 0.0001
T0	29	0.1	18	0.1	
T1	16351	49.7	6017	43.3	
T2	12119	36.8	5152	37.1	
T3	2522	7.7	1368	9.9	
T4	1898	5.8	1329	9.6	
*N*					< 0.0001
N0	19740	60.0	7640	55.0	
N1mi	1325	4.0	438	3.2	
N1	8105	24.6	3823	27.5	
N2	2285	6.9	1069	7.7	
N3	1464	4.4	914	6.6	
M					< 0.0001
M0	30849	93.7	12767	92.0	
M1	2070	6.3	1117	8.0	
TNM					< 0.0001
I	13299	40.4	4744	34.2	
II	12790	38.9	5364	38.6	
III	4760	14.5	2659	19.2	
IV	2070	6.3	1117	8.0	
Surgery					< 0.0001
Yes	30013	91.2	12415	89.4	
No/unknown	2906	8.8	1469	10.6	
Radiotherapy					< 0.0001
Yes	14824	45.0	5830	42.0	
No/unknown	18095	55.0	8054	58.0	
Chemotherapy					< 0.0001
Yes	23535	71.5	10634	76.6	
No/unknown	9384	28.5	3250	23.4	

### Metastatic patterns

Appendix Table 2 summarized the heterogeneity in metastatic patterns between HR + /HER2 + and HR-/HER2 + subgroups among de novo stage IV breast cancer patients. Comparatively, HR + /HER2 + breast cancer metastasizes in bone (62.1% vs 43.4%, *P* < 0.0001) at the initial diagnosis, with a declining incidence of liver (35.0% vs 43.0%, *P* < 0.0001), lung (30.2% vs 35.4%, *P* = 0.011), and brain involvement (6.5% vs 9.4%, *P* = 0.008). Concerning the single-site pattern, de novo metastatic HR + /HER2 + subtype inclined to present bone-only involvement (30.3% vs 15.7%, *P* < 0.0001) and are less manifest as lung-only (10.1% vs 14.9%, *P* < 0.0001) and liver-only metastasis (10.6% vs 16.8%, *P* < 0.0001). Furthermore, there was an increasing incidence of the paired-site pattern, comprising bone and lung (7.3% vs 4.7%, *P* = 0.004) as well as lung and brain (0.2% vs 1.3%, *P* < 0.0001), in addition to the multiple-organic pattern involving bone, liver, and brain (0.5% vs 1.3%, *P* = 0.011).

**Table2:** Comparative analysis of metastatic patterns between HR + /HER2 + and HR-/HER2 + subgroups among de novo stage IV breast cancer patients

Characteristics	HR + /HER2 + (*N* = 2070)		HR-/HER2 + (*N* = 1117)		*P* value
	No	Percent (%)	No	Percent (%)	
Overall					
Bone	1286	62.1	485	43.4	< 0.0001
Liver	724	35.0	480	43.0	< 0.0001
Lung	625	30.2	395	35.4	0.011
Brain	134	6.5	105	9.4	0.008
One site					
Bone	627	30.3	175	15.7	< 0.0001
Liver	219	10.6	188	16.8	< 0.0001
Lung	209	10.1	166	14.9	< 0.0001
Brain	19	0.9	15	1.3	0.281
Two sites					
Bone and liver	239	11.5	110	9.8	0.154
Bone and lung	151	7.3	52	4.7	0.004
Bone and brain	33	1.6	13	1.2	0.356
Liver and lung	60	2.9	46	4.1	0.078
Liver and brain	5	0.2	5	0.4	0.334
Lung and brain	5	0.2	15	1.3	< 0.0001
Three sites					
Bone, liver, and lung	130	6.3	70	6.3	0.988
Bone, liver, and brain	10	0.5	15	1.3	0.011
Bone, lung, and brain	24	1.2	10	0.9	0.589
Liver, lung, and brain	9	0.4	5	0.4	0.958
Four sites					
Bone, liver, lung, and brain	22	1.1	19	1.7	0.139

### Prognostic profiles

An apparent discrepancy was detected in the overall prognosis of the two subgroups (Appendix Fig. 1). Patients with HR-/HER2 + subtype breast cancer had an inferior survival, with a 3-year OS rate of 85.9% (95% CI 85.6–86.2%) and a 5-year OS rate of 78.8% (95% CI 78.3–79.3%), which was significantly decreased than that of patients from HR + /HER2 + subgroup with the a 3-year and 5-year OS rate of 90.5% (95% CI 90.3–90.7%) and 84.2% (95% CI 83.9–84.5%) (*P* < 0.0001) (Appendix Fig. 1a). This kind of distinctive profile remained consistent in the outcome regarding BCSS, which the 3-year and 5-year BCSS rate was 90.5% (95% CI 90.2–90.8%) and 86.1% (95% CI 85.7–86.5%) for HR-/HER2 + subgroup and was 94.6% (95% CI 94.5–94.7%) and 91.2% (95% CI 91.0–91.4%) for HR + /HER2 + subgroup patients, respectively (*P* < 0.0001) (Appendix Fig. 1b). Regarding the HR-specific subtypes, a profound heterogeneity was demonstrated in the prognosis of HER2-positive breast cancer patients with ER + /PgR + , ER + /PgR−, ER−/PgR + , ER−/PgR− subtypes, in which a successively worsening tendency existed in the survival with the 3-year OS rate of 91.8% (95% CI 91.6–92.0%), 87.8% (95% CI 87.4–88.2%), 84.7% (95% CI 83.4–86.0%), 85.9% (95% CI 85.6–86.2%) and 5-year OS rate of 85.8% (95% CI 85.5–86.1%), 80.5% (95% CI 79.9–81.1%), 79.4% (95% CI 77.7–81.1%), 78.8% (95% CI 78.3–79.3%) (*P* < 0.0001). For BCSS, the 3‐year survival rates were 95.7% (95% CI 95.5–95.9%), 92.4% (95% CI 92.1–92.7%), 89.9% (95% CI 88.8–91.0%), 90.5% (95% CI 90.2–90.8%) and 5‐year survival rates were 92.6% (95% CI 92.4–92.8%), 88.3% (95% CI 87.8–88.8%), 85.4% (95% CI 83.8–87.0%), 86.1% (95% CI 85.7–86.5%) of ER + /PgR + , ER + /PgR−, ER−/PgR + , ER−/PgR− groups (*P* < 0.0001), respectively (Appendix Fig. 2a, b). Results of the pairwise comparisons suggested that both the OS and BCSS of ER + /PgR + subtype were significantly favorable than the others (*P* < 0.0001). No statistical significance was detected in OS and BCSS between ER + /PgR− and ER−/PgR + subgroups and in neither OS nor BCSS between ER−/PgR + and ER−/PgR− subtypes (Table S1, 2).

**Fig. 1:**
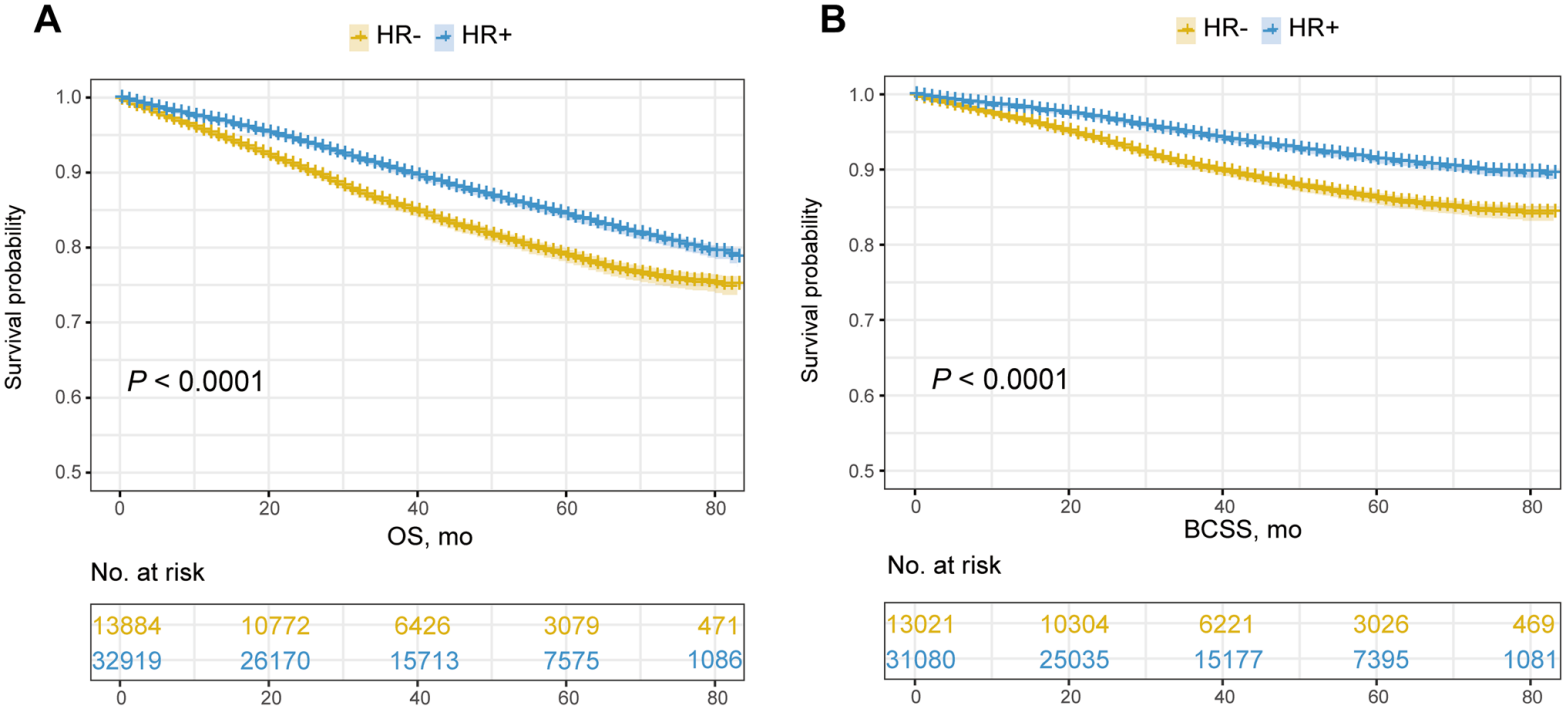
Comparative analysis of OS (**a**) and BCSS (**b**) between HR + /HER2 + and HR-/HER2 + subgroups. *HR* hormone receptor, *HER2* human epidermal growth factor receptor 2, *OS* overall survival, *BCSS* breast cancer-specific survival

**Fig. 2:**
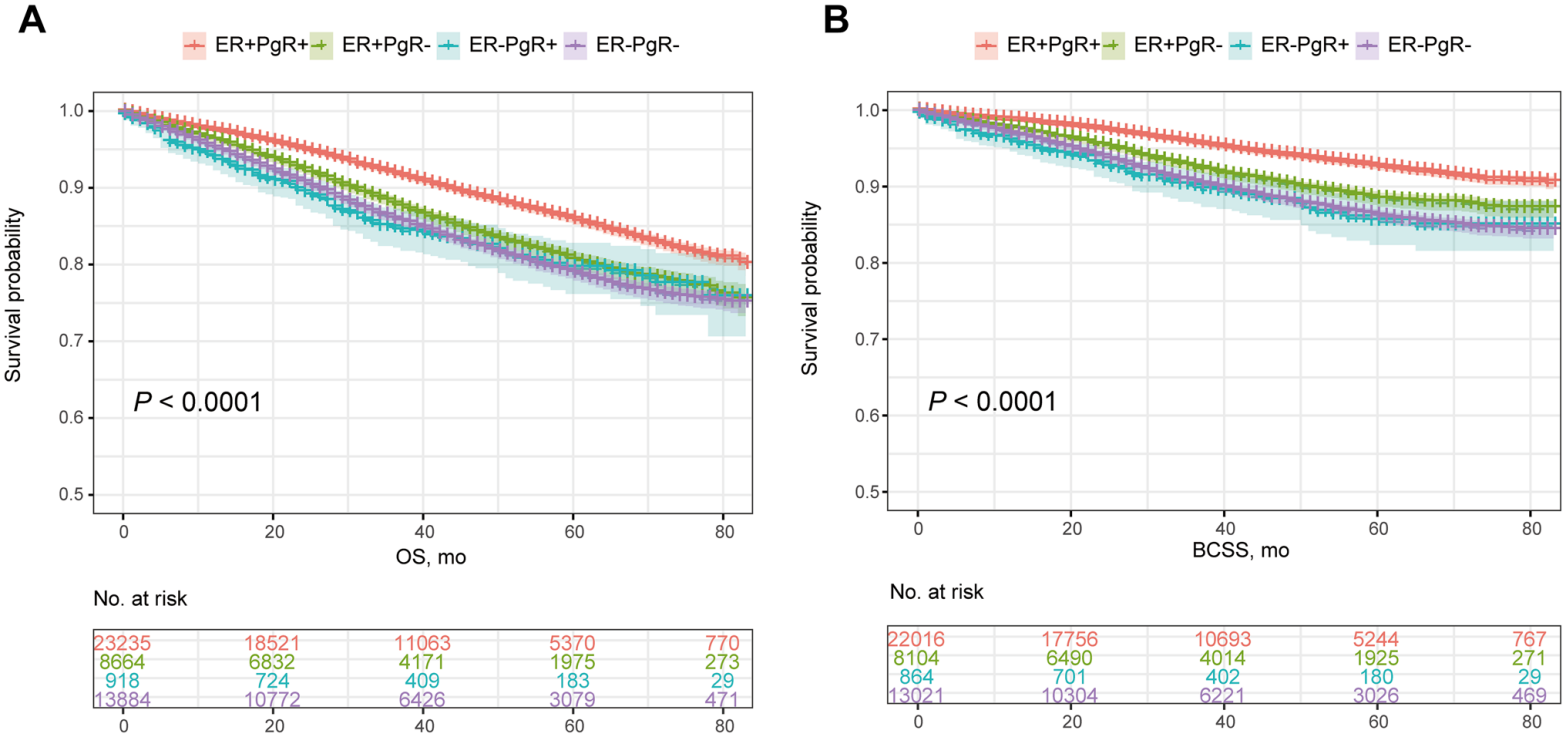
Comparative analysis of OS (**a**) and BCSS (**b**) associated with ER and PgR status. *ER* estrogen receptor, *PgR* progesterone receptor, *OS* overall survival, *BCSS* breast cancer-specific survival

With the performance of PSM analysis, the heterogeneous profile of prognosis kept stable in HER2-positive breast cancer associated with HR status, which both the OS and BCSS were in favor of HR-positive subtype (3-year and 5-year OS, 87.4% vs. 86.0% and 80.0% vs. 78.9%, *P* = 0.015; 3-year and 5-year BCSS, 92.0% vs. 90.6% and 87.3% vs. 86.1%, *P* = 0.0017) (Figure S1a, b). With regard to the prognosis associated with HR-specific subtype, the double-positive subgroup constantly exhibited the utmost favorable prognosis, while a significant divergence existed in both OS and BCSS between ER + /PgR + and ER−/PgR + subtype breast cancer (3-year and 5-year OS, 89.2% vs. 80.8% and 81.9% vs. 74.2%, *P* < 0.0001; 3-year and 5-year BCSS, 93.4% vs. 86.3% and 89.0% vs. 79.5%, *P* < 0.0001) (Figure S2a, b). Of note, the ER−/PgR + subtype tended to present an inferior prognosis, of which the OS (*P* = 0.004 < 0.05/6) and BCSS (*P* = 0.001 < 0.05/6) was greatly shortened than that of ER−/PgR− subtype (Table S3, 4).

Additionally, we investigated the comparative prognosis of the two subgroups of HER2-positive breast cancer associated with the organ-specific involvement (Figure S3). It was denoted that the prognosis of HR + /HER2 + breast cancer patients were superior to that of HR−/HER2 + breast cancer presenting with visceral metastasis (*P* < 0.0001), lung-only disease (*P* = 0.00012), and brain involvement (*P* = 0.0048), while no significant difference was exhibited in patients between the two subgroups with bone-only metastasis, liver-only metastasis, and brain-only metastasis.

## Discussion

In this study, we curated promising impact of the HR expression on the heterogeneous outcomes of clinicopathological characteristics, metastatic patterns, and overall prognosis of HER2-positive breast cancer. To our knowledge, this was the first study that overall discussed the impact of HR status on the clinical characteristics and prognostic profiles of HER2-positive breast cancer based on a large-scale cohort, of which the findings could provide promising evidence for introduction of treatment strategies in clinical practice.

We firstly investigated the potential difference in the clinicopathological characteristics between the two subgroups of HER2-positive breast cancer. In accordance with previous data [15], the proportion of the white race was significantly advantageous in HR + subgroup, while the percentage of the black race was relatively higher in HR- breast cancer patients, which the individual risk factors comprising reproductive history, lactation, physical activity, mammography, and postmenopausal hormone use may explain this kind of ethnic disparity [16]. A consistent discrepancy was also detected in disease features, which HR−/HER2 + breast cancer exhibited a comparatively higher tumor grade and TNM stage, indicative of an increasing aggressiveness and progressive cancer behaviors. This proportion of results were in consistent with previous studies that reported the higher proportion of advanced stage and high-grade tumors among HR-/HER2 + cases when compared to HR + /HER2 + breast cancer patients [7, 11, 17–20]. Concerning treatment options, patients with HR + /HER2 + breast cancer held a climbing opportunity to receive locoregional therapeutics, such as surgery and radiotherapy, yet a less access to systemic delivery, which could be attributed to the distinctive disease factors of the two subgroups observed in the cancer course.

Notably, our investigation provided unique evidence of the associations between HR expression and metastatic patterns of newly diagnosed HER2-positive breast cancer. As expected, patients from HR + /HER2 + subgroup presented an overwhelming frequency of bone involvement with 62.1% of the entire HER2-positive cohort, while HR−/HER2 + tumors tended to metastasize in viscera including liver and lung. This kind of strong correlation between HR status and involved organs in HER2-positive disease was proposed early in 1991 [21] and confirmed in the following studies [11, 12, 22]. With an in-depth understanding of the modulated components in the various subgroups of breast cancer, there had been some studies emerging which could potentially interpret this phenomenon. For instance, the down-regulation of focal adhesion signaling in HR-negative patients was an important contributor of visceral involvement and the absence of Wnt/β-catenin signaling allowed for HR-positive tumors t metastasize in bone [23, 24]. In the current study, brain metastasis was more commonly occurred in the HR-negative population, which was also observed in several studies [11, 12, 22, 25] and could be the promoting profile of the conversely hyperactive Wnt/β-catenin signaling pathway [23]. These results suggest that clinicians should pay more attention to the organs liable to metastasis differing by HR status for de novo stage IV breast cancer patients.

Regarding prognostic profiles, our study revealed a great heterogeneity associated with HR expression in HER2-positive breast cancer, of which the HR + expression was in line with the mortality decrease of both OS and BCSS and this kind of correlation was inherently stable. It is undeniable that adjuvant treatment including targeted and endocrine therapy could affect the prognosis of HER2-positive and HR + /HER2 + breast cancer. Yet, the proportion of patients receiving adjuvant targeted and endocrine therapy were unavailable in SEER database. Trastuzumab, the first humanized monoclonal antibody against HER2, has been officially approved by the FDA for the treatment of HER-2-overexpressing breast cancers in 1998. Several real-world studies [26–31] demonstrated the percentage of adjuvant target therapy based on trastuzumab-containing regimens ranged from 65.6 to 88.8% among HER2 + breast cancer patients in the US. Previous literature has also reported that the majority of HR + patients (63.8–86%) treated with adjuvant endocrine therapy [32–37]. Thereby, we may suppose that patients identified from SEER database have received standard adjuvant treatments. This finding was in accordance with the previous outcomes, in which the HERA trial evaluated 1703 HER2 + early breast cancer patients underwent standard 1-year trastuzumab found a 3-year disease free survival (DFS) of 84.6% in HR + subgroup and 76.4% in HR- subgroup, respectively [38]. Likewise, a study included 3,177 patients with HER2 + breast cancer [39] showed a significantly favorable survival in patients with HR + /HER2 + subgroup compared with HR-/HER2 + subtype with the receipt of standard adjuvant therapeutics.

A potential reason for this sort of prognostic disparity could lie in the multiple therapeutic options in the course of cancer management, especially the contents of medication therapies after recurrence. Given the considerable advances in endocrine therapy, such as fulvestrant, CDK4/6 inhibitors (ribociclib, palbociclib, abemaciclib) and everolimus, the prognosis of HR + metastatic breast cancer has improved immensely [40], which could lead to a superior prognosis on HR + patients. In addition, molecular mechanisms might partly account for the distinct prognosis of HR + /HER2 + and HR−/HER2 + subtype population. Preclinical studies corroborated that PI3K, MAPK, and NOTCH were significantly overexpressed in HR−/HER2 + breast cancer, which could be potentially correlated to therapeutic resistance and lead to the poor survival outcome [41]. Indeed, the specific influence of HR status on the overall prognosis of HER2-positive breast cancer remained controversial [42], and this kind of inconsistency may due to the relatively limited volume of sample size, insufficient follow-up, and the discordance in inclusion criteria. Under this circumstance, this large-scale, population-based study provided strong evidence for the distinctive prognosis of HR-based HER2-poaistive breast cancer population. Considering the relative worse prognosis of HR−/HER2 + patients, physicians may increase the intensity of chemotherapy and targeted therapy such as the combination of trastuzumab and pertuzumab [43], and neratinib following adjuvant trastuzumab-based therapy [44] to reduce relapse rate and enhance survival in clinical practice.

As the mainstay consideration for breast cancer subtypes, the steroid hormone receptors ER and PgR are two critical biomarkers for assessing the intrinsic heterogeneity and introducing multidisciplinary therapeutics [45]. However, to date, the understanding of the clinical significance of ER and PgR status to HER2-positive breast cancer, especially regarding the HR-specific expression patterns, has been poorly investigated. Based on this cohort with extensive enrollment, we suggested that the prognosis of double-positive subtype was the most favorable in the entire population, and ER-negative exerted the foremost impact on the overall prognosis of HER2-positive breast cancer. Consistently, Bae and colleagues [46] indicated that ER + /PgR− and ER−/PgR + breast cancer were in associations with poorer DFS and OS than ER + /PgR + tumors and findings of Rakha et al. [47] adopting 1,944 breast cancer patients suggested that the single-positive patterns, including ER + /PgR− and ER−/PgR + , breast cancer exhibited an increasingly aggressive clinicopathological features than ER + /PgR + subtypes and an opposite profile in comparisons with the ER−/PgR− subtype. In this perspective, physicians could apply individual-based treatment in accordance with the varied HR-specific patterns.

The novel findings of this study lay in that the ER−/PgR + tumor tended to present an inferior prognosis, even to that of ER−/PgR− subtype breast cancer. This finding was in consistent with the previous study that the ER loss exerted a greater effect on the prognosis that PgR loss which could result in a poor survival of breast cancer patients [48]. In the routine practice, ER−/PgR + subtype breast cancer was considered as endocrine-related and would receive endocrine therapy and chemotherapy of which the protocol was with moderate strength given the estimated favorable prognosis. However, Kunc and colleagues [36] contended that patients with ER−/PgR + breast cancer would derive less benefit from the standard endocrine therapy than ER + /PgR + disease instead of chemotherapy. In this perspective, the insufficient therapies could be attributed to the poorer survival in comparisons with the other subtypes. Accordingly, practitioners are supposed to use more caution to introduce reasonable therapeutics towards different HR-specific subtypes of HER2-positive breast cancer. In particular, ER−/PgR + /HER2 + subtype that is relatively insensitive to endocrine therapy, should be treated with higher-intensity chemotherapy and targeted therapy in the adjuvant setting.

Inevitably, our study has several limitations. For starters, potential selection bias introduced by the missing data could not be fully avoided due to the retrospective nature of our study. Also, therapeutic information regarding endocrine therapy and anti-HER2 targeted therapy is not available in this database and these factors accordingly cannot be adjusted for the analysis, thereby leading to potential confounders and deviations in the study. This kind of dilemma also occurs in the absence of a few clinical parameters, such as Ki67 index, ECOG scores, and vascular invasion and part of the prognostic information in terms of recurrence-free and disease-free survival. Furthermore, although the PSM analysis were conducted to reduce the confounding factors, any bias due to the imbalance of the two groups cannot be totally excluded. Finally, the follow-up time could be insufficient by the year in which the complete information of receptor status became available in the SEER database.

## Conclusions

In conclusion, this study elucidated the profound differences in clinical outcomes of HER2-positive breast cancer associated with HR status, existing across the clinicopathological features, metastatic patterns, and overall prognosis. Given the great differences in inherent behaviors and clinical outcomes of this subtype breast cancer, the HR-associated heterogeneity should be fully considered in the course of therapeutic strategies introduction and cancer management for HER2-positive breast cancer in clinical practice.

## Electronic supplementary material

Below is the link to the electronic supplementary material.

Supplementary file1 (DOCX 879 kb)

## Appendices

See Tables 1, 2 and Figs. 1, 2.
