# Supplementary material for: The impact of hormone receptor on the clinical outcomes of HER2-positive breast cancer: a population-based study
Source: Int J Clin Oncol. 2022 Jan 18;27(4):707–16. doi: 10.1007/s10147-022-02115-x (PMC8956538; doi:10.1007/s10147-022-02115-x)

**Supplementary Materials**

**TableS1 Comparative analysis of overall survival between HR+/HER2+ and HR-/HER2+ subgroups**

| ER+P**g**R+ 75.0 (74.7-75.3) |  |  |  |
| --- | --- | --- | --- |
| <0.0001* | ER+P**g**R- 72.1 (71.5-72.6) |  |  |
| <0.0001* | 0.084 | ER-P**g**R+ 70.6 (68.8-72.5) |  |
| <0.0001* | <0.0001* | 0.774 | ER-P**g**R- 70.8 (70.3-71.3) |

*P values indicate statistically significant results

**TableS2 Comparative analysis of breast cancer-specific survival between HR+/HER2+ and HR-/HER2+ subgroups**

| ER+PgR+ 78.9 (78.7-79.1) |  |  |  |
| --- | --- | --- | --- |
| <0.0001* | ER+PgR- 76.5 (76.1-77.0) |  |  |
| <0.0001* | 0.010 | ER-PgR+ 74.6 (73.0-76.2) |  |
| <0.0001* | <0.0001* | 0.582 | ER-PgR- 75.0 (74.6-75.3) |

*P values indicate statistically significant results

**TableS3 Comparative analysis of overall survival between HR+/HER2+ and HR-/HER2+ subgroups after a 1:1 propensity score matching analysis**

| ER+PgR+ 72.8 (72.2-73.3) |  |  |  |
| --- | --- | --- | --- |
| <0.0001 | ER+PgR- 69.3 (68.4-70.2) |  |  |
| <0.0001 | 0.134 | ER-PgR+ 67.4 (64.7-70.1) |  |
| <0.0001 | 0.002* | 0.004* | ER-PgR- 70.9 (70.4-71.3) |

*P values indicate statistically significant results

**TableS4 Comparative analysis of breast cancer-specific survival between HR+/HER2+ and HR-/HER2+ subgroups after a 1:1 propensity score matching analysis**

| ER+PgR+ 76.9 (76.4-77.3) |  |  |  |
| --- | --- | --- | --- |
| <0.0001 | ER+PgR- 74.2 (73.4-74.9) |  |  |
| <0.0001 | 0.023 | ER-PgR+ 71.5 (69.0-73.0) |  |
| <0.0001 | 0.042 | 0.001* | ER-PgR- 75.1 (74.7-75.5) |

*P values indicate statistically significant results

**FigureS1 Comparative analysis of OS (a) and BCSS (b) between HR+/HER2+ and HR-/HER2+ subgroups after a 1:1 propensity score matching analysis**

HR=hormone receptor, HER2=human epidermal growth factor receptor 2, OS=overall survival, BCSS=breast cancer-specific survival


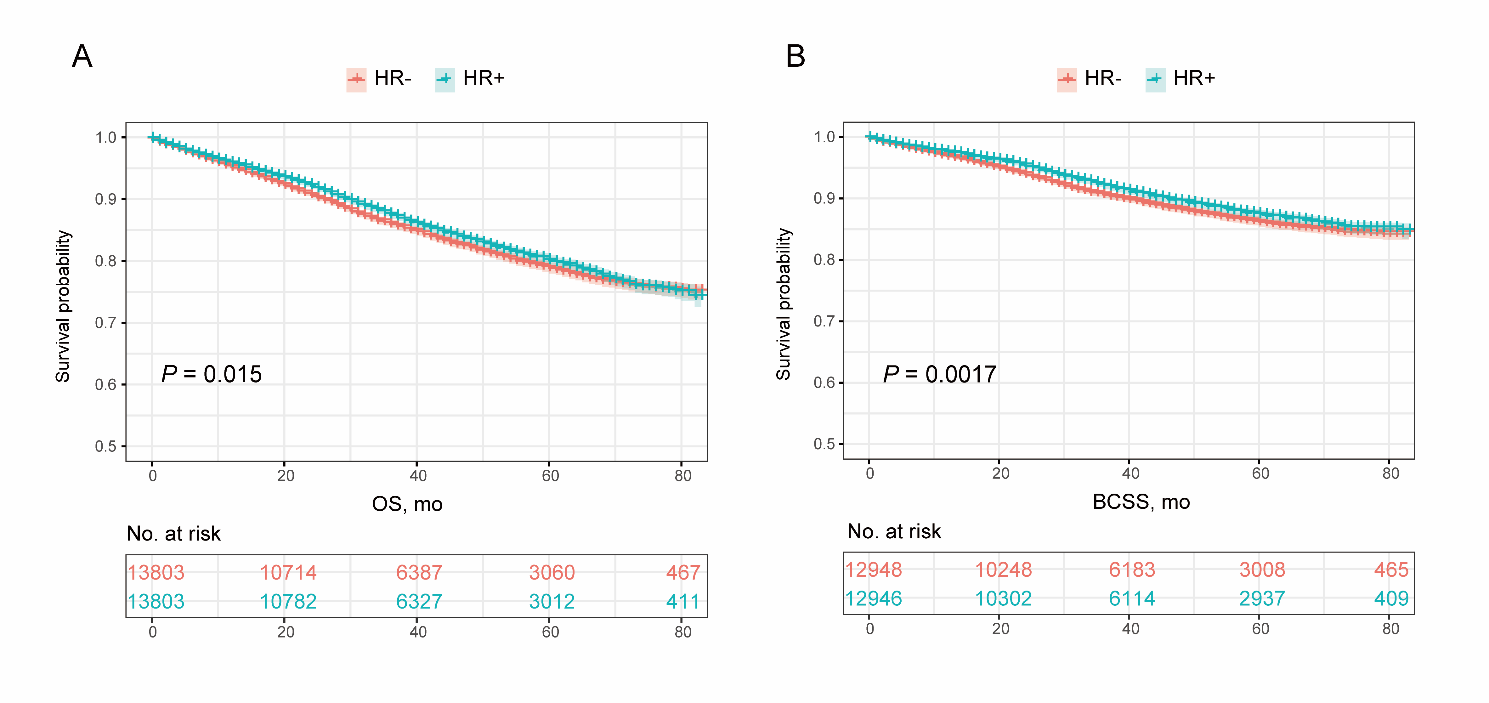


**FigureS2 Comparative analysis of OS (a) and BCSS (b) associated with ER and PgR status after a 1:1 propensity score matching analysis**

**
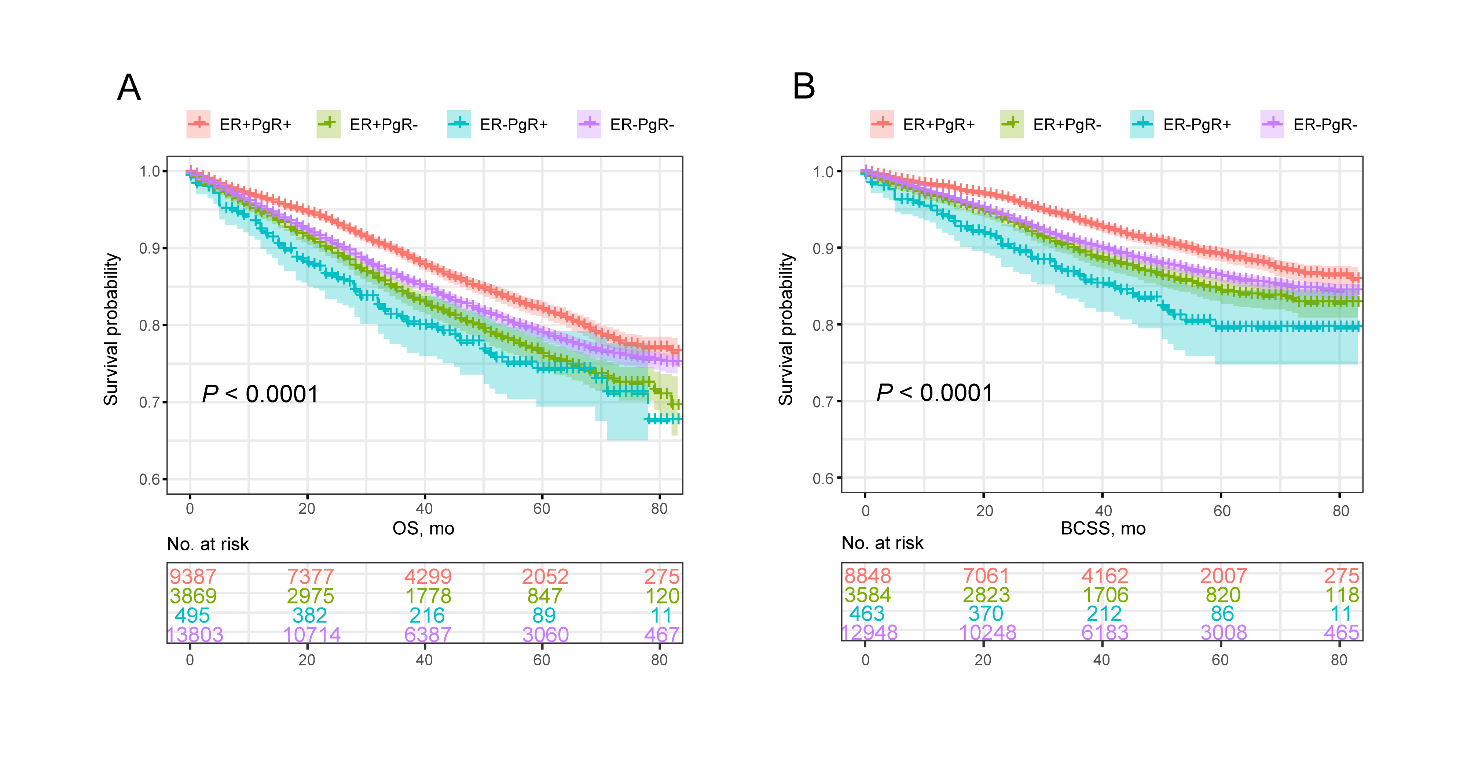
**ER=estrogen receptor, PgR=progesterone receptor, OS=overall survival, BCSS=breast cancer-specific survival

**FigureS3 Comparative analysis of the overall prognosis regarding metastatic patterns including visceral metastasis (a), bone-only metastasis (b), lung-only metastasis (c), liver-only metastasis (d), brain-only metastasis (e) and brain metastasis (f)**


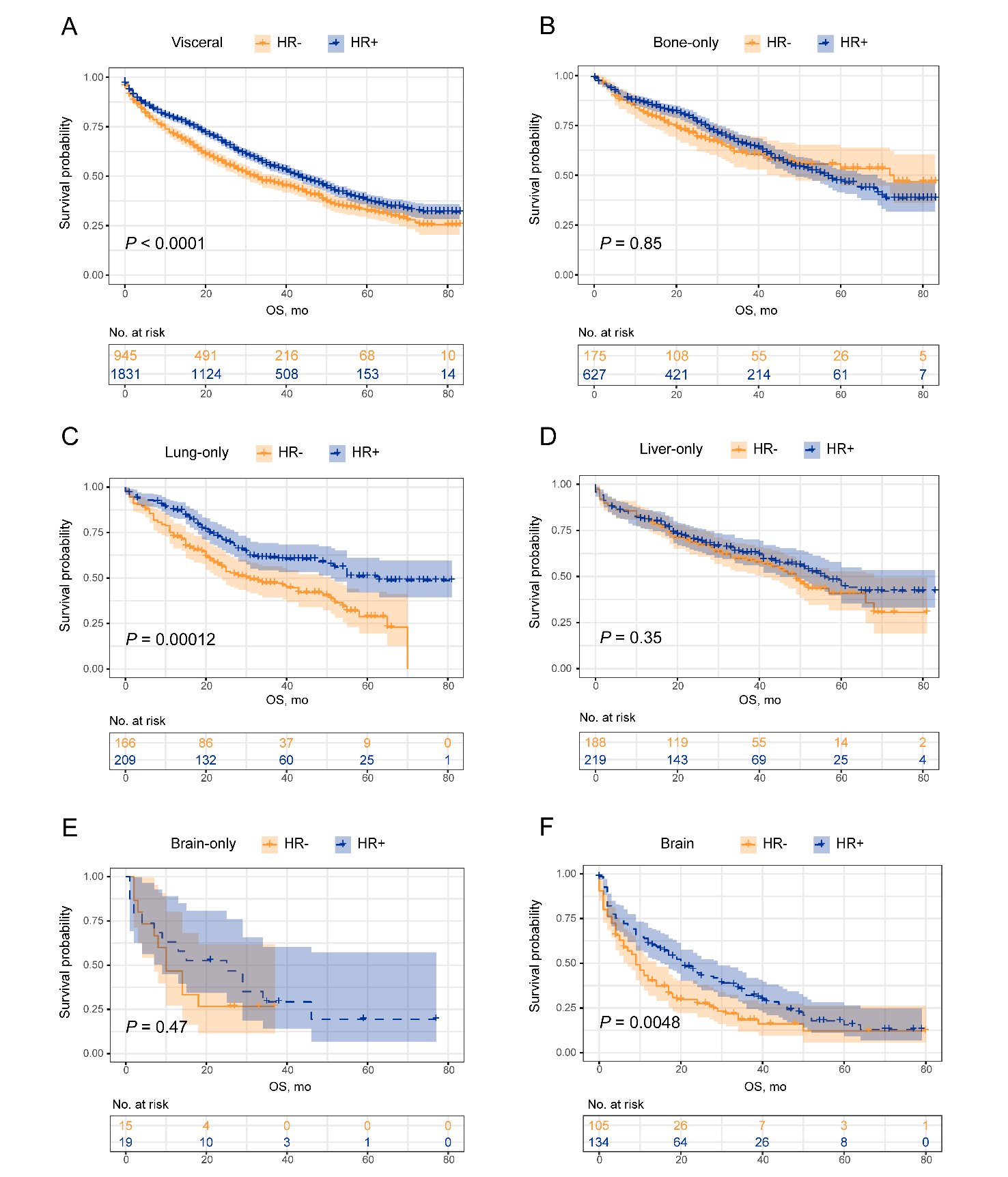

Supplement: Supplementary file 1 — Supplementary file1 (DOCX 879 kb) [file 10147_2022_2115_MOESM1_ESM.docx]
